# Surgical Repair of Morgagni Hernia With Liver Herniation and Rib Fractures in an Adult Following Rhinovirus-induced Coughing

**DOI:** 10.7759/cureus.102530

**Published:** 2026-01-29

**Authors:** Olivia Slogrove, Pouya Nezafati, Sumit Yadav

**Affiliations:** 1 Cardiothoracic Surgery, Mater Private Hospital, Townsville, AUS; 2 Cardiothoracic Surgery, John Hunter Hospital, Newcastle, AUS

**Keywords:** diaphragmatic hernia, liver hernia, morgagni hernia, rib fracture, surgical repair

## Abstract

Morgagni hernias (MH) are rare congenital diaphragmatic defects that often remain undiagnosed until adulthood due to their subtle or nonspecific clinical presentation. While asymptomatic in many cases, MHs may occasionally lead to visceral herniation; among these, atraumatic liver herniation, especially in the absence of prior thoracoabdominal trauma, is exceptionally uncommon and represents a notable diagnostic and clinical challenge. We report the case of a 62-year-old male patient with no history of trauma who presented with right-sided pleuritic chest pain following a rhinovirus infection. Initial chest CT demonstrated a lateral chest wall hernia with herniation of the right lower lobe and a widened intercostal space. Conservative inpatient management was initially pursued, but the patient re-presented with persistent respiratory symptoms and right upper abdominal pain. Repeat CT imaging revealed progression of the hernia with new involvement of the right hepatic lobe, gallbladder, and omentum, alongside displaced fractures of the eighth and ninth ribs and disruption of the intercostal musculature. Surgical repair was performed via a right video-assisted thoracoscopic surgery (VATS)-assisted thoracotomy. Operative findings included a 15 × 10 cm diaphragmatic defect anterior to the caval hiatus, herniated liver, chronic empyema, and rib fractures. The defect was repaired using a 15 × 25 cm Symbotex™ mesh (Medtronic plc, Galway, Ireland), and rib fractures were stabilised using segmented STRACOS 3D clips (MedXpert GmbH, Eschbach, Germany). The patient recovered uneventfully and was discharged from the ICU on postoperative day three. This case illustrates a rare instance of progressive Morgagni hernia with visceral and hepatic herniation triggered by virus-induced coughing in the absence of trauma. It highlights the diagnostic challenges associated with atypical thoracoabdominal symptoms and emphasizes the importance of maintaining a high index of suspicion. Early imaging and timely surgical intervention are critical to prevent serious complications and achieve favourable outcomes in such complex presentations.

## Introduction

Morgagni hernias (MH) are rare congenital diaphragmatic defects, accounting for approximately 4-5% of all congenital diaphragmatic hernias (CDH) [1]. They arise from incomplete fusion of the septum transversum and costal arches, resulting in a weakness in the sternocostal triangle through which abdominal contents, such as omentum, colon, and in more severe cases, liver, stomach, or small bowel, may herniate into the thoracic cavity [2].

Although MHs are present from birth, they often remain asymptomatic and undetected until adulthood. In adults, diagnosis is frequently delayed due to vague or nonspecific symptoms, most commonly respiratory or upper abdominal in nature [2]. Factors that increase intra-abdominal pressure, such as obesity, forceful coughing, chronic constipation, or trauma, may contribute to enlargement of the defect and promote herniation, eventually leading to clinical symptoms [1,3-5]. Atraumatic liver herniation is sparsely reported in the literature and may occur with or without biochemical evidence of hepatic impairment [5,6]. When left untreated, such a herniation carries the risk of serious complications, including laceration, necrosis, rupture, or strangulation of the herniated viscera [7].

Surgical repair is the recommended treatment for MH, even in asymptomatic cases, due to the risk of visceral compromise [3,4]. The choice of approach is tailored to patient characteristics. Thoracic approaches, such as median sternotomy, thoracotomy, or thoracoscopy, provide superior access for right-sided defects and facilitate dissection from intrathoracic structures. Abdominal approaches, including laparotomy and laparoscopy, allow for evaluation and reduction of intra-abdominal contents and inspection of the contralateral diaphragm [8]. Robotic-assisted laparoscopic repair has also been described, offering enhanced visualization and instrument control, particularly in complex or recurrent cases [9].

Herein, we report a unique case of an MH triggered by rhinovirus-induced coughing, resulting in progressive herniation of the liver, gallbladder, and omentum, accompanied by rib fractures and intercostal muscle disruption, ultimately necessitating surgical intervention following failure of conservative management.

## Case presentation

A 62-year-old male patient with no history of thoracoabdominal trauma presented with a four-day history of severe right-sided pleuritic chest pain, flu-like symptoms, and persistent vigorous coughing with right upper quadrant abdominal tenderness. Initial laboratory investigations revealed a mild elevation in inflammatory markers, with a negative septic screen. Rhinovirus was detected on polymerase chain reaction (PCR) testing, and there was no biochemical evidence of hepatic dysfunction.

**Figure 1:**
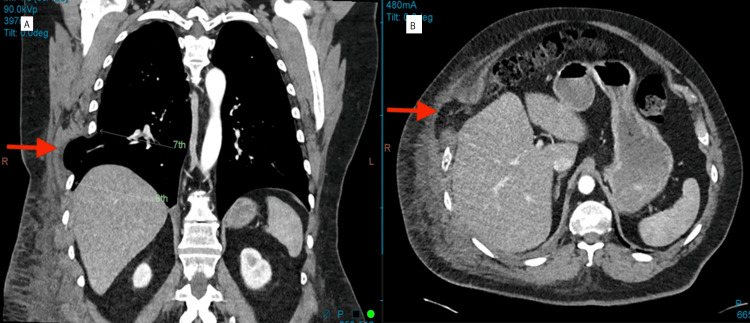
Right chest wall hernia with rib splaying Chest wall hernia of right lower lobe and omental fat — (A) Coronal view showing right lateral wall herniation with rib splaying; (B) Axial view demonstrating herniated omental fat and lung

A chest computed tomography (CT) scan demonstrated a 6 cm widening of the interspace between the right seventh and eighth ribs, with a secondary lung herniation containing the right lower lobe and pleura (Figure 1) and an unremarkable CT scan of the abdomen. The patient was conservatively managed for rhinovirus and remained an inpatient for 12 days before sub-optimal resolution of symptoms was achieved, and was subsequently discharged home. 

**Figure 2:**
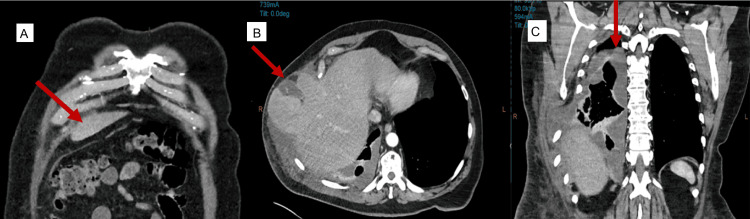
Liver and gallbladder herniation and empyema (A) Coronal view showing herniation of liver and gallbladder through chest wall; (B) Axial view of same herniation; (C) Coronal view of the chest demonstrating herniation of the right hepatic lobe into the thoracic cavity through an anterior diaphragmatic defect at the right cardiophrenic region, supporting the intraoperative diagnosis of a Morgagni hernia.

**Figure 3:**
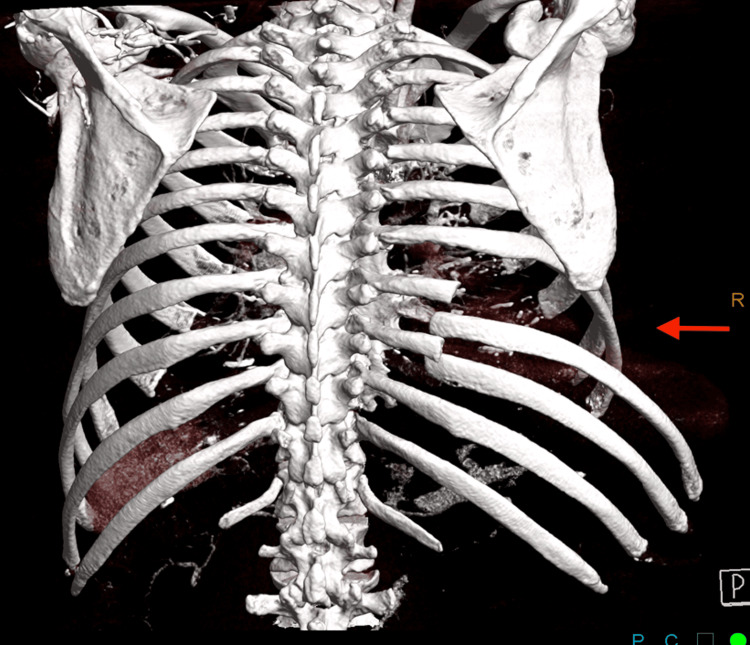
Posterior rib fractures with displacement Posterior rib reconstruction demonstrating displaced fractures of the left eighth and ninth ribs.

The patient re-presented five days post-discharge with ongoing right-sided pleuritic chest pain, persistent non-productive cough, and worsening abdominal pain. Repeat laboratory investigations, including full blood count and liver function tests, were within normal limits. Follow-up contrast-enhanced CT of the chest demonstrated interval progression of the right lateral thoracic wall hernia, now involving herniation of the right hepatic lobe, gallbladder, and omentum into the pleural cavity (Figure 2A, 2B). Coronal CT (Figure 2C) demonstrated hepatic herniation through the anterior diaphragm into the thoracic cavity, supporting a diaphragmatic rather than intercostal origin. Additionally, new findings included displaced fractures of the right eighth and ninth ribs, with an increased intercostal space measuring approximately 10 cm, and disruption of the adjacent intercostal musculature (Figure 3).

The patient subsequently underwent operative intervention via a right posterolateral video-assisted thoracoscopic surgery (VATS)-assisted thoracotomy. Intraoperative findings included dense adhesions between the right upper and lower lobes and the parietal pleura of the chest wall, in addition to a chronic loculated empyema cavity. A large anterior diaphragmatic defect, measuring approximately 15 × 10 cm, was identified in the right parasternal cardiophrenic region. Intraoperative thoracoscopic assessment confirmed a true anterior parasternal diaphragmatic defect within the sternocostal triangle (foramen of Morgagni), with herniation of the right hepatic lobe through the defect into the thoracic cavity. Fractures were noted at the posterior ends of the eighth and ninth ribs, with associated structural instability.

**Figure 4:**
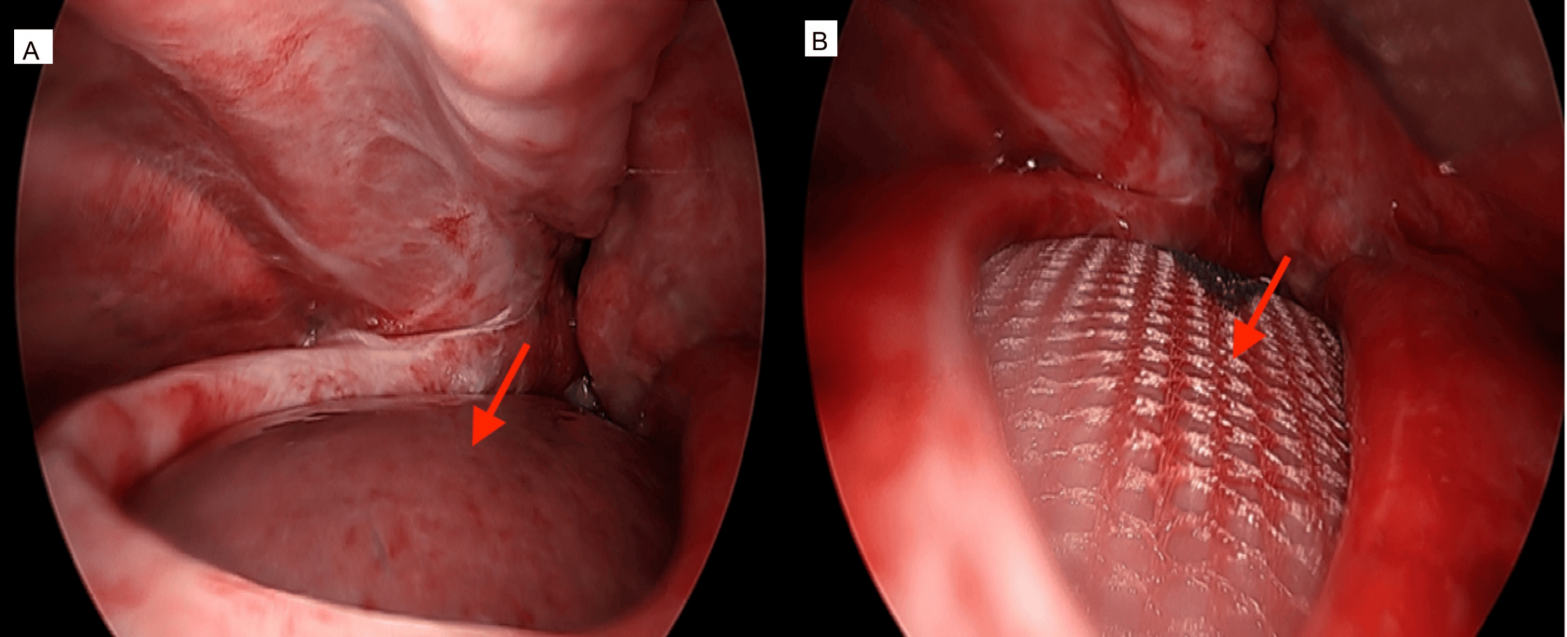
Morgagni hernia with mesh repair (A) Herniated liver through Morgagni defect on coronal view; (B) Mesh repair of diaphragmatic defect after reduction.

Following meticulous adhesiolysis and thorough debridement of the pleural cavity, the herniated liver (Figure 4A) was reduced into the abdominal compartment. The diaphragmatic defect was repaired using a 15 × 25 cm Symbotex™ mesh (Medtronic plc, Galway, Ireland) (Figure 4B), which was anchored anteriorly with interrupted 2-0 Ethibond sutures (Ethicon, Inc., Raritan, New Jersey, United States), while the remainder of the defect was closed using 2-0 pledgeted sutures to ensure tension-free closure and reinforcement. Concomitant rib fractures were stabilized using segmented STRACOS 3D rib clips (MedXpert GmbH, Eschbach, Germany). The patient was extubated in the immediate postoperative period and transferred to the intensive care unit for monitoring. He remained hemodynamically stable and was stepped down to the general ward on postoperative day three without complications.

## Discussion

MHs are a diagnostic pitfall across clinical and radiological platforms, which may contribute to delayed diagnoses and the omission of appropriate therapeutic interventions [3]. Surgical fixation is necessary to avoid vascular compromise of herniated contents, including incarceration, strangulation, and necrosis [9,10]. Efficacy of repair extends to traumatic and atraumatic patient cohorts, as well as asymptomatic [4,11-13].

Unlike Bochdalek hernias, which are posterolateral congenital diaphragmatic defects that typically present in neonates with respiratory distress due to pulmonary hypoplasia, up to 50% of MHs remain asymptomatic and are often diagnosed incidentally in adulthood [14]. The presence of a hernia sac, formed by the parietal peritoneum encapsulating the herniated abdominal contents, may limit direct compression or irritation of thoracic structures and has therefore been proposed as a factor contributing to the frequently asymptomatic nature of MHs [7].

Anatomically, MHs are more commonly right-sided, while left-sided and bilateral presentations are less frequent. This is thought to be due to the protective effect of the heart and pericardial attachments, which reinforce the left-sided sternocostal triangle and reduce the likelihood of herniation on that side [1,8]. MHs are typically small anterior retrosternal defects, but progressive enlargement can occur, often complicating timely diagnosis [3]. Elevated intra-abdominal pressures, such as those induced by forceful coughing, obesity, or straining, can lead to further widening of the defect across the hemidiaphragm [3]. We favour this mechanism as the likely cause of hepatic and gallbladder herniation through the foramen of Morgagni in our patient, triggered by rhinovirus-associated coughing and exacerbated by pre-existing central obesity.

Chest X-ray has been reported to detect MHs in up to 71% of cases, although CT remains the most sensitive and specific diagnostic modality [8,12]. On CT, herniated omentum may appear as a fat-density mass in the retrosternal space, while bowel-containing hernias present as air- or fluid-filled loops within the thoracic cavity [4,10]. These radiological features are non-specific and may be misinterpreted as lipomas, hiatal hernias, or lateral chest wall defects [8,15]. This diagnostic overlap underscores the importance of early cross-sectional imaging in patients with unexplained thoracoabdominal symptoms, particularly when initial imaging is inconclusive. Prompt and accurate identification of MHs can expedite appropriate surgical referral and prevent complications associated with delayed diagnosis.

Definitive management of MH is surgical repair, which offers excellent long-term outcomes and low recurrence rates [13]. The choice of approach should be tailored to patient-specific factors, including hernia size, content, laterality, presence of adhesions or infection, and overall clinical condition [12]. Both open and minimally invasive techniques, including thoracotomy, laparotomy, thoracoscopy, and laparoscopy, have demonstrated comparable perioperative morbidity and mortality in adults [12].

In our case, a thoracotomy was selected due to the complexity of the intra-thoracic pathology, which included a large diaphragmatic defect, dense pleural adhesions, displaced rib fractures with intercostal muscle disruption, and chronic loculated empyema. The empyema likely developed secondary to chronic extrapleural inflammation from progressive diaphragmatic and chest wall disruption, with subsequent secondary pleural involvement. While thoracoscopic techniques can be effective for selected cases, including adhesiolysis and diaphragmatic repair, the extent and combination of pathologies in the current patient rendered a minimally invasive approach less feasible. Open thoracotomy enabled comprehensive management, including safe reduction of herniated hepatic and biliary structures, thorough debridement of infected pleural fluid, mesh reinforcement of the diaphragmatic defect, and stabilization of rib fractures. This approach ensured optimal exposure, procedural efficiency, and reduced the risk of intraoperative complications.

Thoracotomy is particularly advantageous for right-sided MHs, offering direct access to pleural and mediastinal structures and allowing for durable reconstruction [3-8]. While it may limit evaluation of the contralateral diaphragm, its utility remains high in anatomically complex or recurrent cases. Thoracoscopic repair remains a valuable minimally invasive option in patients with less extensive disease burden, but its application may be limited in scenarios requiring multi-component intervention or involving dense tissue planes [2].

In emergency presentations involving bowel obstruction, visceral ischemia, or acute respiratory compromise, an open transabdominal approach via laparotomy is often favoured [8,12]. This allows for rapid reduction of herniated contents, direct assessment of bowel viability, repair of associated intra-abdominal pathology, and evaluation of the contralateral diaphragm, particularly when bilateral hernias are suspected [3]. Beyond emergencies, laparotomy is also indicated in cases involving large or complex hernias, dense adhesions, or when anatomical distortion precludes safe minimally invasive access.

In contrast, laparoscopy is generally reserved for stable patients with uncomplicated MHs, particularly when the diaphragmatic defect is small to moderate in size, and the herniated contents are easily reducible [8]. Compared to open laparotomy, laparoscopy offers several perioperative advantages, including reduced postoperative pain, shorter hospital stay, faster return to function, and lower overall morbidity [3,8]. Importantly, the transabdominal approach aligns well with the anterior anatomical location of Morgagni defects, allowing direct access for effective reduction of herniated viscera and safe mesh placement. It also enables simultaneous evaluation of both hemidiaphragms, which is valuable in identifying occult or bilateral defects.

Emerging robotic-assisted transabdominal repairs provide enhanced 3D visualization, improved instrument dexterity, and greater ergonomic precision, making them an appealing option in select elective cases, although their availability and longer operative setup times currently limit widespread adoption [9].

Currently, there is no clear consensus on the routine use of mesh versus primary suture repair for MHs, largely due to the limited number of comparative studies and the rarity of the condition [2]. However, mesh reinforcement is generally considered beneficial in select situations, particularly in cases involving large defects (typically ≥20-30 cm²), attenuated or thinned diaphragmatic tissue, or when significant tension is anticipated at the repair site [3,8]. The decision to use mesh remains at the discretion of the operating surgeon and is often guided by intraoperative findings.

Similarly, the resection of the hernia sac remains a topic of debate. While sac removal may facilitate better visualization and repair, it has been associated with potential complications such as pneumomediastinum, injury to the phrenic nerve, or damage to the superior epigastric vessels [5,8]. Furthermore, there is currently insufficient evidence to definitively link sac resection with either increased or decreased recurrence risk [8].

## Conclusions

This case illustrates an uncommon presentation of MH in an adult, triggered by viral-induced coughing and complicated by progressive visceral herniation, chest wall disruption, and chronic empyema. It emphasizes the importance of including MH in the differential diagnosis of atypical thoracoabdominal symptoms, even in the absence of trauma. Timely diagnosis through appropriate imaging is essential to prevent serious complications. While minimally invasive techniques are preferred in selected cases, this case highlights the value of an open thoracotomy approach in complex scenarios requiring concurrent hernia reduction, mesh repair, pleural debridement, and rib fixation. A tailored surgical strategy based on anatomical and clinical factors remains crucial for achieving durable and safe outcomes.

## References

[1] Horton JD, Hofmann LJ, Hetz SP (2008). Presentation and management of Morgagni hernias in adults: a review of 298 cases. Surg Endosc.

[2] Sanford Z, Weltz AS, Brown J, Shockcor N, Wu N, Park AE (2018). Morgagni hernia repair: a review. Surg Innov.

[3] Mohamed M, Al-Hillan A, Shah J, Zurkovsky E, Asif A, Hossain M (2020). Symptomatic congenital Morgagni hernia presenting as a chest pain: a case report. J Med Case Rep.

[4] Albasheer O, Hakami N, Ahmed AA (2023). Giant Morgagni hernia with transthoracic herniation of the left liver lobe and transverse colon: a case report. J Med Case Rep.

[5] Harne PS, Mukherjee S, Shepherd Z (2020). Atraumatic intercostal and intrathoracic liver herniation related to influenza A. ACG Case Rep J.

[6] Mutluoglu M, Van Robaeys F, Gryspeerdt S, De Smet K (2022). Non-traumatic intrathoracic herniation of the caudate lobe. BMJ Case Rep.

[7] Lee SY, Kwon JN, Kim YS, Kim KY (2018). Strangulated Morgagni hernia in an adult: synchronous prolapse of the liver and transverse colon. Ulus Travma Acil Cerrahi Derg.

[8] Ağalar C, Atila K, Arslan NÇ, Derici ZS, Bora S (2019). Adult morgagni hernia: a single center experience of 5 cases and review of literature. Turk J Surg.

[9] Phillips J, Brigode WM, Svahn J (2022). Rapid evolution of a Morgagni hernia with herniation of the left hepatic lobe: case report and review of the literature. J Surg Case Rep.

[10] Kuikel S, Shrestha S, Thapa S, Maharjan N, Kandel BP, Lakhey PJ (2021). Morgagni hernia in adult: a case report. Int J Surg Case Rep.

[11] Bendinelli C, Martin A, Nebauer SD, Balogh ZJ (2012). Strangulated intercostal liver herniation subsequent to blunt trauma. First report with review of the world literature. World J Emerg Surg.

[12] Fatima SZ, Khadeeja S, Naqvi SM (2023). An unusual presentation of adult Morgagni hernia complicated with anuria. J Med Surg Public Health.

[13] Ben-Yaacov A, Menasherov N, Bard V (2020). Repair of a recurrent symptomatic hernia through the foramen of Morgagni: a case study and review of the literature. J Surg Case Rep.

[14] Loong TP, Kocher HM (2005). Clinical presentation and operative repair of hernia of Morgagni. Postgrad Med J.

[15] Makis W, Rush C (2011). Liver herniation through the foramen of Morgagni: a pitfall in oncologic F-18 FDG PET/CT evaluation of the anterior mediastinum. Clin Nucl Med.

